# Data on iron oxide core oil-in-water nanoemulsions for atherosclerosis imaging

**DOI:** 10.1016/j.dib.2017.10.059

**Published:** 2017-10-26

**Authors:** Geoffrey Prévot, Stéphane Mornet, Cyril Lorenzato, Tina Kauss, Laurent Adumeau, Alexandra Gaubert, Julie Baillet, Philippe Barthélémy, Gisèle Clofent-Sanchez, Sylvie Crauste-Manciet

**Affiliations:** a Univ. Bordeaux, INSERM, U1212, CNRS UMR 5320, ARNA, ARN: Régulations Naturelle et Artificielle, ChemBioPharm, F-33000 Bordeaux, France; b CNRS, Univ. Bordeaux, ICMCB, UPR 9048, F-33600 Pessac, France; c Univ. Bordeaux, CNRS UMR 5536, CRMSB, Centre de Résonance Magnétique des Systèmes Biologiques, F-33000 Bordeaux, France

## Abstract

The data presented in this article are related to the publication entitled “Iron oxide core oil-in-water nanoemulsion as tracer for atherosclerosis MPI and MRI imaging” (Prévot et al., 2017) [1]. Herein we describe the synthesis and the characteristics of the Superparamagnetic Iron Oxide Nanoparticles (SPION) loaded inside nanoemulsions (NEs). Focus was set on obtaining SPION with narrow size distribution and close to superparamagnetic limit (20 nm) in order to reach a reasonable magnetic signal. Nanoparticles (NPs) of three different sizes were obtained (7, 11 and 18 nm) and characterized using transmission electron microscopy (TEM), X-ray diffraction (XRD), vibrating sample magnetometer (VSM), diffuse reflectance infrared Fourier transform (DRIFT) and thermogravimetric analysis (TGA). SPION were coated with oleic acid (OA) in order to load them inside the oily core of NEs droplets. SPION loaded NEs were magnetically sorted using MACS® MS Column (Miltenyi Biotec) and iron quantification was performed by UV-spectrometry measurements.

**Specifications Table**

**Table:** 

Subject area	*Chemistry and material science*
More specific subject area	*Nanoparticle synthesis and characterisation*
Type of data	*Synthesis protocols and figures*
How data was acquired	*Nanoparticles characteristics were obtained by Transmission Electron Microscopy (TEM), X-ray diffraction (XRD), Vibrating Sample Magnetometer (VSM), Diffuse Reflectance Infrared Fourier Transform (DRIFT) and Thermogravimetric Analysis (TGA)*
	*Iron quantification was performed by UV-spectrometry measurements*
Data format	*Analysed*
Experimental factors	*Synthesis of maghemite nanoparticles (γ-Fe*_*2*_*O*_*3*_*) by coprecipitation or thermolysis of ferric oleate*
Experimental features	*Evaluation of maghemite nanoparticles sizes and magnetic behaviour*
Data source location	*Bordeaux, France*
Data accessibility	*The data is provided with this article*

**Value of the data**

• The data presented in this paper describe two synthesis processes in order to obtain SPION from 3 different sizes 7, 11 and 18 nm.
• SPION physico-chemical properties were characterized by TEM, XRD, DRIFT, TGA and VSM.
• NEs magnetic sorting process using MACS® MS Column (Miltenyi Biotec) and iron quantification data are also described.
• These data will be helpful for the scientific community that work with magnetic nanosystems.

## 1 Data

**Fig. 1:**
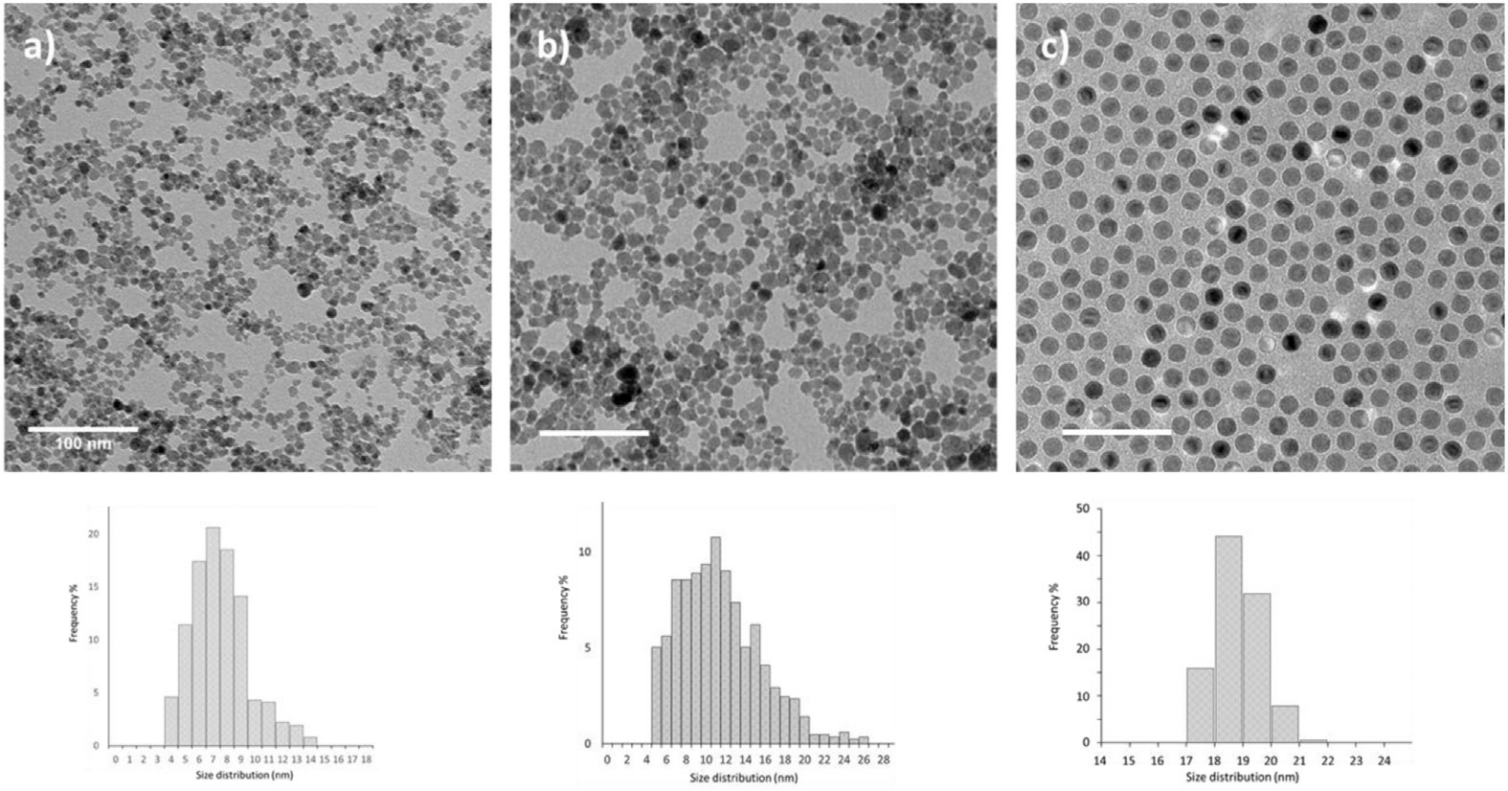
TEM micrographs of NPs of 7 (a), 11 (b) and 18 nm (c) in diameter. The values of average sizes and standard deviations are listed in Table 1.

**Fig. 2:**
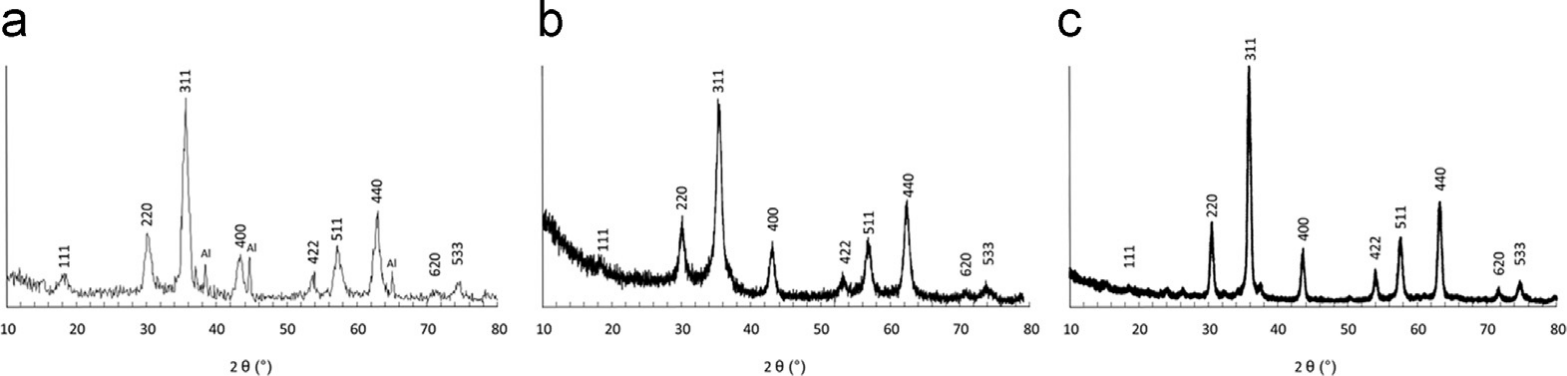
XRD spectra of NPs of 7 (a), 11 (b) and 18 nm (c) in diameter. The values of the average crystallite sizes are listed in Table 1.

**Fig. 3:**
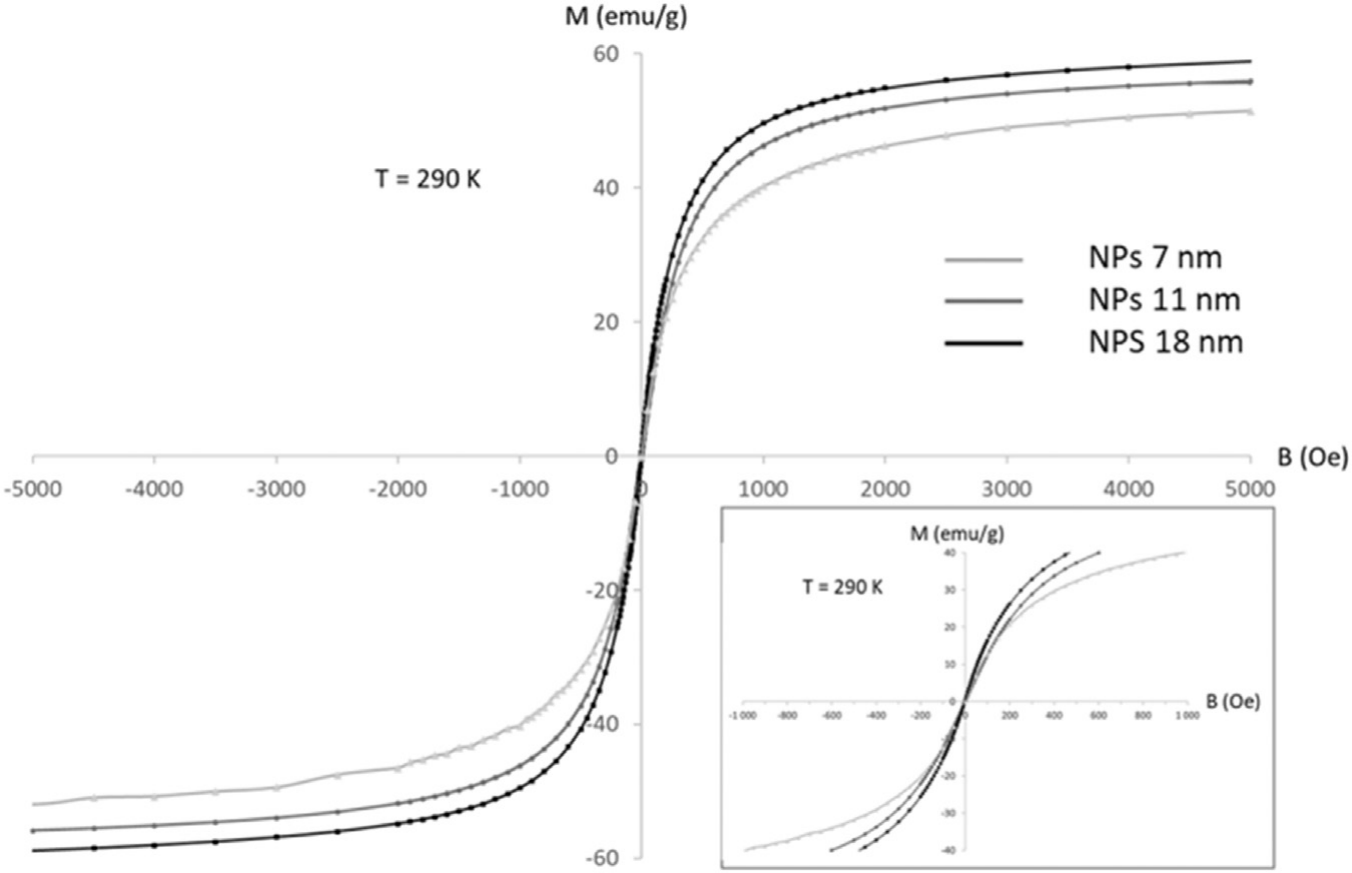
Magnetic behaviour of each NPs used in this study. In insert, detail of the magnetization curves for the field range [−1000 Oe;1000 Oe] showing differences in magnetic susceptibility (slope at the origin of the curves).

**Fig. 4:**
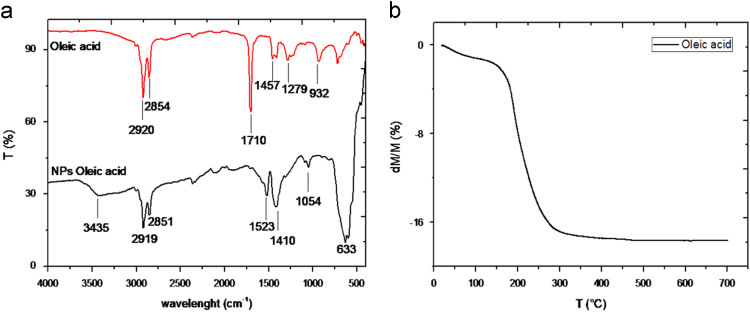
a) Diffuse Reflectance IR transform spectra of oleic acid (OA) and NPs-7nm-OA samples. The strong IR bands at 2919 and 2851 cm^-1^ are assigned to asymmetrical and symmetrical stretching modes of the alkyl chains (CH_2_), while the band at 1410 cm^-1^ is attributed to deformation vibration (δCH_2_). In addition to these bands, supplementary shoulder at 1279 cm^-1^ corresponding to C-O vibration attests the presence of OA to NPs surface. The band at 633 cm^-1^ is characteristic to Fe-O-Fe vibrations. b) Thermogram of NPs-7nm-OA sample that displays a mass loss of 15.9% between 120 °C and 350 °C attributed to the degradation of OA ligand. This mass loss corresponds to a grafting density of 2 molecules/nm².

**Fig. 5:**
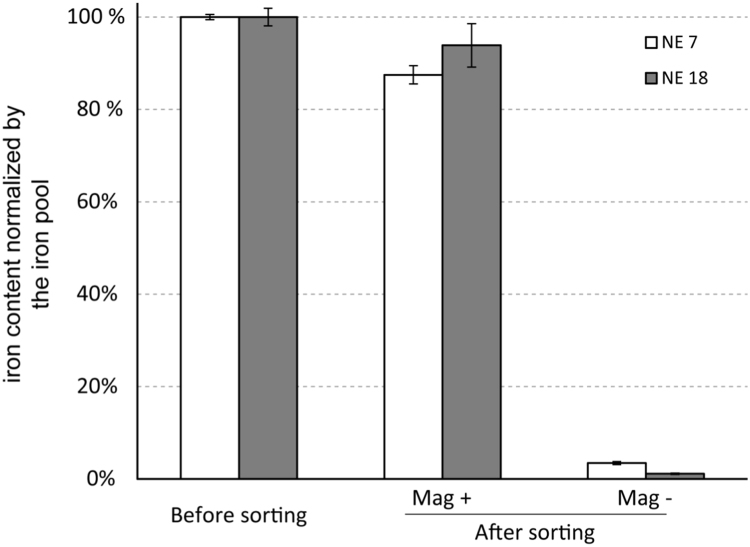
Iron quantification before and after magnetic sorting using MACS® MS Column performed on nanoemulsions loaded with NPs of 7 nm (NE 7) and nanoemulsions loaded with NPs of 18 nm (NE 18).

The dataset of this articles provides information about the engineering of hydrophobic SPION and their loading inside NEs for imaging purpose [1]. Fig. 1, Fig. 2 and Table 1 display the size characteristics of the NPs obtained by TEM and XRD. Fig. 3 displays the magnetic behavior of the 3 different NPs obtained by VSM. NPs coating was assessed by DRIFT and TGA analysis (Fig. 4). Fig. 5 shows the iron quantification of NEs before and after magnetic sorting.

**Table 1:** Values of average sizes (D), standard deviations (σ) obtained by TEM and crystallite sizes by XRD analysis. The average crystallite sizes were obtained from the EVA software after assessment of the integral width of the most intense peak (311) and the Scherrer formula $\left(t=\frac{\lambda }{\beta \cos ϴ}\right)$.

Sample	NPs (γ-Fe_2_O_3_) 7 nm	NPs (γ-Fe_2_O_3_) 11 nm	NPs (γ-Fe_2_O_3_) 18 nm
D (TEM)	7.5	11.3	18.3
σ (TEM)	2.5	3.6	0.5
D (XRD)	6.9	9.4	14.9

## 2 Experimental design, materials and methods

### 2.1 Magnetic nanoparticles synthesis

Maghemite NPs of 7 nm diameter were synthesized by alkaline coprecipitation of ferrous and ferric salts as previously described [2]. NPs of 11 nm were obtained using size sorting procedure according to Sandre and al. [3]. Hydrophobization of these NPs was performed by complexation of OA on the SPION surface according to the procedure described by Van Ewijk et al. [4]. For 18 nm sized NPs, the synthesis derived from a slightly modified procedure previously described by Sun et al. [5] and hydrophobic NPs are directly obtained.

### 2.2 Characterizations of NPs

TEM micrographs were performed with a Philips CM120 microscope operating at 120 kV and captured with Ultra scan USC1000 2k×2k camera. Assessments of NPs sizes were performed with the Digital Micrograph software from Gatan on more than 300 NPs.

XRD on dried powders were performed with a PANalytical X'pert PRO MPD diffractometer in Bragg–Brentano (θ–θ) geometry Cu Kα radiation (*λ*=1.5418 Å) to check the crystallographic structure and to assess the crystallite size (size of the coherent domains of diffraction). Magnetic measurements were performed with a VSM MicroSence FCM-10 at 290 K for a field H varying to ±5000 Oe. DRIFT spectroscopy was performed with a Bruker IFS Equinox 55 FTIR spectrometer (signal averaging 32 scans at a resolution of 4 cm^-1^) equipped by a selector Graseby Specac diffuse reflection cell (Eurolabo, France).

TGA were assessed using a Tag2400 thermobalance from Setaram (Caluire-et-Cuire, France). TGA measurements were effected by applying a first temperature cycle from 25 °C to 650 °C by increasing heat of 2 °C/min under O_2_.

### 2.3 Iron quantification

Iron was quantified by UV-spectrometry measurements. Samples were prepared following this procedure: ethanol was used to break the emulsion. After 10 min centrifugation at 14,000 g, precipitates were washed with ethanol and centrifuged again. Then the precipitate was dissolved into HCl (2%)/HCOOH, in order to dissolve the maghemite NPs into Fe^3+^ ions. These ions react with KSCN to produce a red compound allowing the iron concentration determination by spectrometry measurements at 466 nm.
