# Supplementary material for: Data on iron oxide core oil-in-water nanoemulsions for atherosclerosis imaging
Source: Data Brief. 2017 Oct 26;15:876–81. doi: 10.1016/j.dib.2017.10.059 (PMC5675726; doi:10.1016/j.dib.2017.10.059)
Supplement: Supplementary file 1 — Supplementary material [file mmc1.pdf]

Conflicts of Interest Statement

Manuscript title: Data on iron oxide core oil-in-water nanoemulsions for atherosclerosis imaging

The authors whose names are listed immediately below certify that they have NO affiliations with or involvement in any organization or entity with any financial interest (such as honoraria; educational grants; participation in speakers' bureaus; membership, employment, consultancies, stock ownership, or other equity interest; and expert testimony or patent-licensing arrangements), or non-financial interest (such as personal or professional relationships, affiliations, knowledge or beliefs) in the subject matter or materials discussed in this manuscript.

Author names:

Geoffrey Prévot,
Stéphane Mornet,
Cyril Lorenzato,
Tina Kauss,
Laurent Adumeau,
Alexandra Gaubert
Julie Baillet
Philippe Barthélémy
Gisèle Clofent-Sanchez
Sylvie Crauste-Manciet

The authors whose names are listed immediately below report the following details of affiliation or involvement in an organization or entity with a financial or non-financial interest in the subject matter or materials discussed in this manuscript. Please specify the nature of the conflict on a separate sheet of paper if the space below is inadequate.

Author names:

This statement is signed by all the authors to indicate agreement that the above information is true and correct (*a photocopy of this form may be used if there are more than 10 authors*):

Author's name (typed)	Author's signature	Date
PREVOT		13 th October 2017
MORNET		October 11th 2017
LORENZATO		10th October 2017
KAUSS		Oct 12th 2017
ADUMEAU		11th October 2017
GAUBERT		oct. 12h 2017
BAILLET		oct. 12h 2017
BARTHELEMY		oct 11t 2017
CLOFENT-SANCHEZ		10th October 2017
CRAUSTE-MANCIET		october 10th 2017
